# Effectiveness of underwater treadmill rehabilitation following femoral head and neck ostectomy in dogs: A prospective controlled pilot study

**DOI:** 10.14202/vetworld.2026.3621-3634

**Published:** 2026-08-17

**Authors:** Nichanan Maneeganondh, Naruepon Kampa, Thanikul Srithunyarat, Pongsatorn Tuchpramuk, Phimrawee Ngampring, Parichat Naknil, Somphong Hoisang

**Affiliations:** 1 Veterinary Teaching Hospital, Faculty of Veterinary Medicine, Khon Kaen University, Khon Kaen 40002, Thailand.; 2 Department of Veterinary Structural and Clinical Interventional Sciences, Faculty of Veterinary Medicine, Khon Kaen University, Khon Kaen 40002, Thailand.; 3 Faculty of Veterinary Sciences, Mahasarakham University, Maha Sarakham 44150, Thailand.; 4 Undergraduate Veterinary Students, Faculty of Veterinary Medicine, Khon Kaen University, Khon Kaen 40002, Thailand.

**Keywords:** aquatic rehabilitation, canine rehabilitation, femoral head and neck ostectomy, force plate gait analysis, hydrotherapy, orthopedic rehabilitation, peak vertical force, underwater treadmill

## Abstract

**Background and Aim:**

Femoral head and neck ostectomy (FHO) is a widely performed salvage procedure for canine coxofemoral disorders; however, postoperative recovery is frequently limited by reduced weight bearing, persistent lameness, muscle atrophy, and restricted hip mobility. Although underwater treadmill (UWTM) rehabilitation has been used for various orthopedic conditions, objective evidence supporting its effectiveness after FHO remains limited. This study aimed to evaluate the effectiveness of a standardized 10-session UWTM rehabilitation protocol on functional recovery in dogs after FHO, using clinical and objective gait assessment measures.

**Materials and Methods:**

A prospective controlled pilot study was conducted using 10 client-owned dogs that underwent unilateral FHO. Dogs were allocated to either a UWTM rehabilitation group (n = 5) or a non-UWTM control group (n = 5). One dog in the UWTM group was withdrawn due to force plate noncompliance, leaving four treatment and five control dogs to complete the 12-week follow-up. Rehabilitation consisted of 10 weekly UWTM sessions initiated approximately 3 weeks after surgery. Clinical evaluations included lameness and pain scores, thigh circumference, and force plate kinetic gait analysis. Peak vertical force (PVF) normalized to body weight (BW) served as the primary objective outcome measure. Assessments were performed at postoperative weeks 4, 8, and 12.

**Results:**

Dogs receiving UWTM rehabilitation demonstrated significant improvements in functional recovery over time. Lameness scores decreased significantly between postoperative months 1 and 3 (mean difference = 2.00 points, p < 0.05), while PVF of the operated limb increased significantly by 23.66% of BW between months 1 and 3 (p < 0.05). Interlimb PVF asymmetry also decreased significantly in the UWTM group. In contrast, the control group showed significant improvement only in lameness score by month 3, whereas PVF did not change significantly throughout the study. Pain scores and thigh circumference did not differ significantly between groups over time. No serious treatment-related adverse events were observed during the rehabilitation protocol.

**Conclusion:**

A standardized 10-session UWTM rehabilitation protocol may accelerate postoperative functional recovery following FHO by improving limb weight bearing and reducing lameness without treatment-related complications. Although these preliminary findings support UWTM as a promising adjunctive rehabilitation strategy after FHO, the small sample size and pilot design limit generalizability. Larger randomized controlled trials with extended follow-up are warranted to establish optimal rehabilitation protocols and confirm long-term clinical benefits.

## INTRODUCTION

Canine hydrotherapy, particularly rehabilitation using an underwater treadmill (UWTM), is an established therapeutic exercise modality in veterinary medicine, widely used to facilitate functional recovery in dogs with orthopedic and neurologic disorders [1, 2]. UWTM rehabilitation enhances muscle strength, improves mobility, increases joint range of motion (ROM), and reduces both acute and chronic pain, thereby promoting earlier restoration of normal locomotion [2, 3]. Its therapeutic benefits are primarily attributed to the unique physical properties of water, including buoyancy, viscosity, hydrostatic pressure, and relative density, which collectively reduce joint loading, support body weight (BW), facilitate controlled limb movement, and improve patient comfort during exercise [1]. Consequently, UWTM has become an increasingly valuable component of postoperative rehabilitation protocols aimed at improving functional outcomes while minimizing mechanical stress on healing tissues [4, 5].

Femoral head and neck ostectomy (FHO) is a well-established salvage orthopedic procedure performed to eliminate pain, crepitus, and abnormal bone-on-bone contact within the coxofemoral joint in dogs affected by conditions such as hip luxation, femoral head and neck fractures, severe osteoarthritis, and avascular necrosis [6–8]. Although FHO effectively relieves pain and substantially improves quality of life, restoration of normal limb function remains challenging during the postoperative period. Quantitative gait analyses have demonstrated that dogs undergoing FHO exhibit significantly reduced static weight bearing on the operated limb compared with the contralateral limb (15.6% versus 20.3% of BW) [9]. Persistent unloading of the affected limb commonly results in muscle atrophy, restricted hip joint mobility, altered gait mechanics, and prolonged circumferential asymmetry between the operated and contralateral limbs [9, 10]. These functional impairments often necessitate structured postoperative rehabilitation to facilitate recovery. Integrating UWTM into postoperative management may accelerate functional restoration by promoting earlier controlled weight bearing while reducing excessive mechanical loading of the healing limb [11]. Current rehabilitation guidelines recommend initiating UWTM once consistent voluntary limb use has been established, typically within 2–3 weeks after surgery, to optimize muscle recruitment and functional recovery [12].

Objective evaluation of postoperative recovery is essential for determining the effectiveness of rehabilitation interventions following FHO. Previous studies have assessed recovery using subjective lameness scoring, pain assessment, thigh circumference measurements [4, 9], and kinetic gait analysis. Among these methods, force plate analysis of peak vertical force (PVF) is considered the gold standard for objectively quantifying limb weight bearing and functional gait recovery in dogs because of its excellent accuracy, reproducibility, and sensitivity to clinically meaningful changes [13–15]. Accordingly, these parameters were selected as the primary outcome measures in the present study.

Although early land-based physiotherapy has been reported to improve postoperative recovery following FHO [16], evidence supporting the effectiveness of UWTM rehabilitation remains limited. To date, no prospective controlled study has objectively evaluated a standardized UWTM rehabilitation protocol following FHO using force plate-derived kinetic gait analysis in conjunction with conventional clinical assessments. Furthermore, previous reports have primarily focused on subjective clinical outcomes or the application of hydrotherapy in dogs with chronic orthopedic or neurologic disorders rather than specifically investigating postoperative functional recovery after FHO. Consequently, the magnitude of improvement in objective weight bearing, clinical lameness, pain, and muscle recovery associated with UWTM remains poorly defined. Addressing this evidence gap is important for establishing evidence-based rehabilitation protocols that optimize postoperative recovery and improve clinical decision-making in canine orthopedic practice.

Therefore, this prospective controlled pilot study aimed to evaluate the effectiveness of a standardized 10-session UWTM rehabilitation protocol following FHO in client-owned dogs using both objective kinetic gait analysis and clinical outcome measures. Specifically, postoperative recovery was assessed through PVF, lameness score, pain score, and thigh circumference over a 12-week follow-up period. We hypothesized that dogs receiving UWTM rehabilitation would demonstrate significantly greater improvements in functional limb use, enhanced weight bearing capacity, and superior overall postoperative recovery than dogs managed without UWTM rehabilitation.

## MATERIALS AND METHODS

### Ethical approval

This prospective, convenience-sampled, controlled pilot study was approved by the Institutional Animal Care and Use Committee, Khon Kaen University, Khon Kaen, Thailand (Approval No. IACUC-KKU-71/66; approved on May 18, 2023). The study was conducted and reported in accordance with the Animals in Research: Reporting *In Vivo* Experiments (ARRIVE) 2.0 guidelines. Throughout the study, all dogs remained under the care and ownership of their respective owners. Before enrollment, each owner received a detailed explanation of the study objectives, experimental procedures, anticipated benefits, and potential risks, after which written informed consent was obtained. Owners were also provided with standardized postoperative home-care instructions, including leash walking, restriction of running and jumping activities, administration of prescribed antibiotics and non-steroidal anti-inflammatory drugs (NSAIDs), and adherence to scheduled follow-up visits for wound evaluation, radiographic examination, and lameness assessment.

### Study period and location

The study was conducted between May and December 2023 at the Veterinary Teaching Hospital, Faculty of Veterinary Medicine, Khon Kaen University (VTH-KKU), Khon Kaen, Thailand. Client-owned dogs that underwent unilateral FHO during the study period were prospectively enrolled. Before surgery, all dogs underwent comprehensive physical and orthopedic examinations, hematological and serum biochemical analyses, and radiographic evaluation. Standard orthogonal radiographs were obtained to confirm unilateral hip luxation or femoral head and neck fractures before surgical intervention [17].

### Study design

This prospective, convenience-sampled, controlled pilot study included client-owned dogs that had undergone unilateral FHO. Postoperative orthopedic and radiographic examinations were performed to confirm satisfactory surgical outcomes and the absence of postoperative complications before enrollment. Owing to feasibility constraints and the limited study duration, convenience sampling was used while adhering to the recommended minimum and maximum numbers of animals for experimental studies. Sample size was determined using the resource equation approach rather than conventional power analysis, which is an accepted method for animal-based research involving limited sample sizes [18, 19].

**Figure 1:**
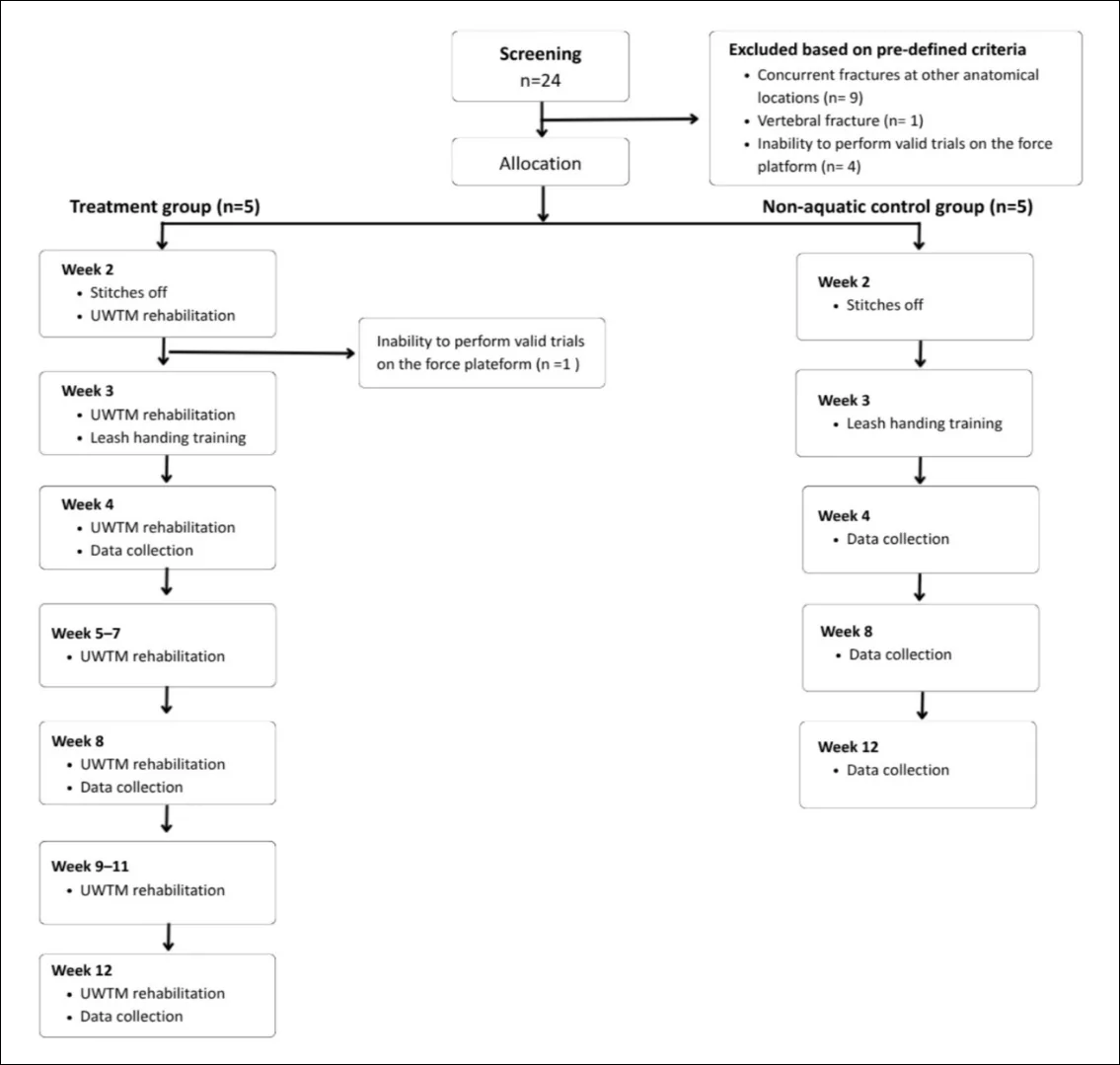
CONSORT-style flow diagram illustrating the study design, screening, group allocation, timeline of rehabilitation, and participant follow-up. One dog in the UWTM treatment group was withdrawn at Week 3 due to force plate non-compliance. UWTM = Underwater treadmill.

A total of 24 client-owned dogs that underwent unilateral FHO were initially screened for eligibility (Figure 1). Fourteen dogs were excluded according to the predefined exclusion criteria, including concurrent fractures at other anatomical sites (n = 9), vertebral fracture (n = 1), and poor leash compliance during gait assessment (n = 4). Consequently, 10 dogs met the eligibility criteria and were allocated by convenience sampling to either the UWTM rehabilitation group (n = 5) or the control group (n = 5), which received no formal physical rehabilitation. Group allocation was performed alternately according to each owner's willingness and ability to attend the scheduled rehabilitation sessions.

To minimize assessment bias, all outcome evaluations were performed by an experienced observer who was not involved in treatment administration. The study commenced on the day of surgery (week 0) and continued until week 12. Although all enrolled dogs were intended to complete the 12-week follow-up period, one dog in the UWTM group was withdrawn at the second follow-up visit (week 3) due to repeated noncompliance during force plate gait analysis. Consequently, four dogs in the UWTM group and five dogs in the control group completed the study, and only these dogs were included in the final analyses.

Dogs were eligible for inclusion if they were at least 6 months of age, weighed >10 kg, were of any breed or sex, had undergone unilateral FHO, and showed no evidence of systemic disease. Eligible dogs were also required to remain free of orthopedic or neurologic complications throughout the study period. Dogs were excluded if they demonstrated a shortened strike phase resulting in an inability to obtain valid force plate recordings or were unable to traverse the force platform consistently. Additional exclusion criteria included unilateral hip luxation secondary to pelvic fractures, concurrent fractures at other anatomical locations, lumbosacral instability, a previous history of orthopedic surgery involving the ipsilateral limb, postoperative surgical site infection, newly developed systemic disease, or neurologic deficits during the study. (Figure 1)

### Surgical procedure

An intravenous catheter was placed in the cephalic vein for administration of Lactated Ringer's solution (3–5 mL/kg/h; General Hospital Products, Pathum Thani, Thailand). Preemptive analgesia was achieved with morphine sulfate (0.5 mg/kg intramuscularly; The Government Pharmaceutical Organization, Bangkok, Thailand), administered 15–20 minutes before anesthetic induction. Dogs were subsequently premedicated with diazepam (0.2 mg/kg intravenously; Diapine; Atlantic Laboratories Corp., Bangkok, Thailand) and prophylactic cefazolin (25 mg/kg intravenously; Cefaben®; L.B.S. Laboratory Corp., Bangkok, Thailand). General anesthesia was induced with propofol (approximately 5–6 mg/kg intravenously, titrated to effect; Profol™; Baxter Pharmaceuticals India Pvt. Ltd., Gujarat, India) to facilitate endotracheal intubation and was maintained with isoflurane (1%–2%; Isoflurane USP; Piramal Critical Care, Bethlehem, PA, USA) delivered in 100% oxygen. Before skin closure, carprofen (4–4.4 mg/kg subcutaneously; Rimadyl®; Zoetis manufacturing & research Spain S.L., Vall de Bianya, Girona, Spain) was administered for perioperative analgesia.

All FHO procedures were performed by a single Thai board-certified veterinary surgeon (SH) with more than 15 years of experience in small animal orthopedic surgery, thereby eliminating inter-operator variability. A standard craniolateral surgical approach was used as previously described [6]. Following completion of the ostectomy, the deep gluteal muscle and subcutaneous tissues were closed separately using 2-0 polydioxanone (PDS II; Ethicon, Raritan, NJ, USA). The skin was routinely closed with 2-0 nylon (Ethilon; Ethicon) in a simple interrupted pattern. No intraoperative complications were encountered in any dog. Immediate postoperative radiographs were obtained and evaluated by the operating surgeon to confirm complete excision of the femoral head and neck. Successful ostectomy was defined by a smooth osteotomy line extending from the medial aspect of the greater trochanter to the proximal femoral shaft without residual bony prominences or spurs.

Postoperative pain was monitored using the Glasgow Composite Measure Pain Scale–Short Form (CMPS-SF) [20]. Rescue analgesia consisting of morphine sulfate (0.3–0.5 mg/kg intramuscularly every 4–6 hours) was planned for dogs with pain scores >5/20; however, none of the enrolled dogs required additional analgesic intervention. Following hospital discharge, all dogs received oral cephalexin (25 mg/kg every 12 hours; Sporidin®; Sun Pharma, Dewas, India) together with carprofen (4–4.4 mg/kg every 24 hours; Rimadyl®; Zoetis, Parsippany, NJ, USA) for 7 days.

### Study protocol and clinical assessment

All rehabilitation procedures and clinical evaluations were conducted at the VTH-KKU rehabilitation facility, which was equipped with a UWTM, computerized gait analysis equipment, and a force plate system (Advanced Mechanical Technology®, AMTI Model OR6-6, Watertown, MA, USA). Two force plates (40 × 60 cm each) were installed sequentially along the center of an 8-m walkway to permit kinetic gait assessment. Force plate data were acquired and analyzed using ToMoCo-FPm software (Toso System Inc®, Saitama, Japan; version 12.02).

During the immediate postoperative period, surgical wounds were examined daily for evidence of complications, including erythema, edema, discharge, or wound dehiscence. Skin sutures were removed 14 days after surgery, and wound healing was reassessed at that time. Radiographic examinations were subsequently performed at scheduled follow-up visits to monitor postoperative progression.

**Figure 2:**
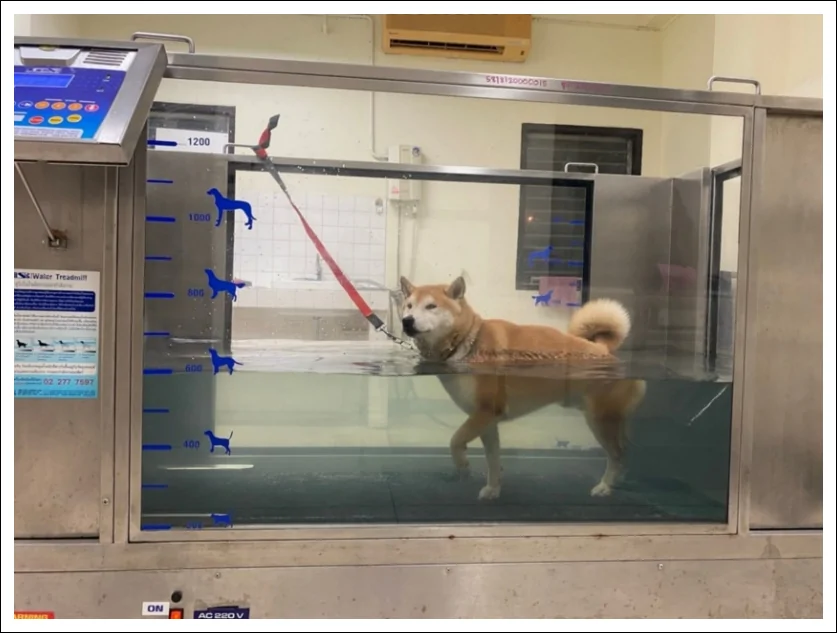
Underwater treadmill rehabilitation session in a dog following unilateral femoral head and neck ostectomy, illustrating limb positioning and water level during the active phase of the rehabilitation protocol.

The UWTM rehabilitation program consisted of a structured three-phase protocol adapted from Lee [21] and Alves [22], comprising initial, active, and cool-down phases, performed once weekly for 10 consecutive sessions (Table 1, Figure 2). This rehabilitation protocol has not previously been evaluated in dogs following FHO. Rehabilitation was initiated during the third postoperative week, with the first session performed at a mean of 21.75 ± 0.96 days after surgery. Water temperature was maintained between 25°C and 28°C throughout all sessions. During the initial phase, water depth was adjusted to the level of the greater trochanter to maximize buoyancy and reduce weight bearing forces. Water depth was gradually decreased to the level of the stifle joint during the cool-down phase to increase active joint movement and muscular effort. Treadmill speed was initially set at 0.22 m/s, increased to 0.50 m/s during the active phase, and subsequently reduced to 0.22 m/s during the cool-down phase. Progression through the rehabilitation protocol was criteria-based, with dogs advancing only after demonstrating comfortable weight bearing at the prescribed water depth and treadmill speed without evidence of fatigue or discomfort.

Safety was continuously monitored during each rehabilitation session, throughout rest intervals, and for 20–30 min after exercise completion. Sessions were terminated immediately if dogs exhibited signs of distress, pain, vocalization, panic-like behavior, or acute lameness. If increased lameness or decreased weight bearing was observed relative to the pre-exercise baseline, rescue analgesia consisting of oral carprofen (4–4.4 mg/kg every 24 hours) was administered for 3–5 days as clinically indicated [23].

**Figure 3:**
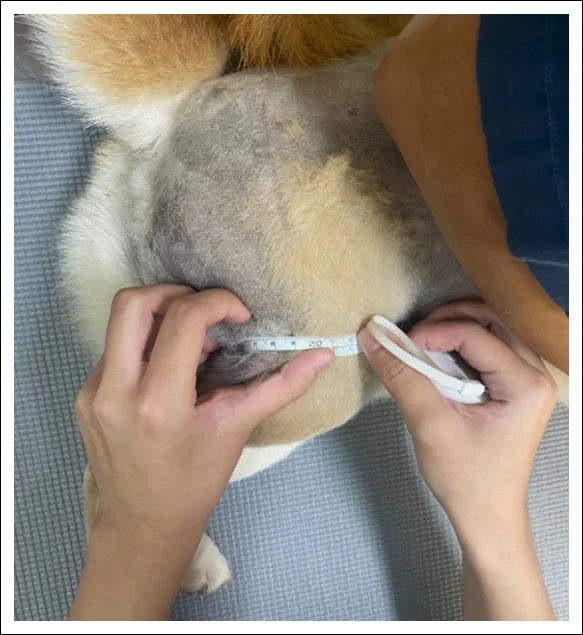
Measurement of thigh circumference in a dog positioned in relaxed lateral recumbency. Circumference was measured using a flexible measuring tape positioned perpendicular to the long axis of the femur at 70% of the femoral length measured distally from the greater trochanter.

Between postoperative weeks 3 and 12, all dogs underwent weekly clinical evaluations that included behavioral observations, thigh circumference measurements [24], assessment of weight bearing during trotting, and lameness evaluation [25]. Comprehensive data collection was performed at postoperative weeks 4, 8, and 12 by a single experienced veterinarian (NM) who was blinded to group allocation. Subjective lameness and pain were assessed using validated modified numeric rating scales ranging from 0 to 4 [26]. Detailed scoring criteria are presented in Tables 2 and 3. Thigh circumference was measured with dogs positioned in relaxed lateral recumbency (Figure 3) at 70% of the femoral length measured from the greater trochanter to the lateral femoral condyle, as previously described [8].

**Table 1:** Underwater treadmill rehabilitation protocol.

**Phase**	**Activity duration**	**Rest duration**	**Repetitions**
Initial phase	45 seconds	2 minutes	2–3 cycles
Active phase	3–6 minutes	2 minutes	3 cycles
Cool-down phase	45 seconds	2 minutes	2–3 cycles
Total exercise time	21–25 minutes	—	—

**Table 2:** Standardized lameness scoring system used for gait assessment at a trot.

**Score**	**Description**
0	No lameness; normal weight bearing throughout all strides
1	Mild, subtle lameness with partial weight bearing
2	Obvious lameness with partial weight bearing
3	Obvious lameness with intermittent weight bearing
4	Complete non-weight bearing lameness

**Table 3:** Pain scoring system based on behavioral responses during joint palpation.

**Score**	**Description**
0	No pain on joint palpation
1	Mild pain; palpation completed without restraint
2	Moderate pain; palpation completed with obvious discomfort
3	Severe pain; palpation could not be completed because of resistance
4	Pain was too severe to permit palpation without restraint or sedation

### Outcome measures

**Figure 4:**
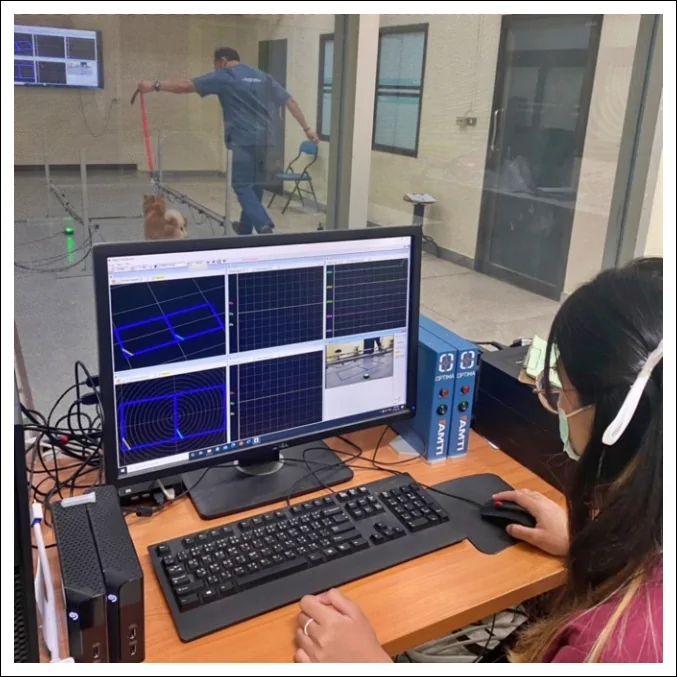
Force plate kinetic gait analysis performed in a dog trotting at a controlled speed (1.7–2.2 m/s) along an 8-m walkway. Ground reaction forces generated by the ipsilateral hindlimb during force plate contact were recorded and analyzed to calculate peak vertical force.

Objective kinetic gait analysis was performed using the force plate system (Figure 4). Before commencement of the study, the designated handler (PT) completed standardized training for 2 weeks to ensure consistent leash handling and trotting speed throughout data collection. Each dog was trotted along the 8-m walkway at a controlled velocity of 1.7–2.2 m/s with acceleration maintained within ±0.5 m/s². A trial was considered valid when the dog trotted in a straight line with a consistent head position, without leash tension, and the ipsilateral hindlimb made complete contact with the force plate. Trials that failed to satisfy these criteria were discarded, and additional trials were performed until three valid recordings were obtained for each dog.

PVF was calculated using ToMoCo-FPm software (Toso System Inc®) and normalized to BW according to the following equation:

## PVF (% BW) = [PVF (N)/BW (N)] × 100

The mean PVF from the three valid trials was used for statistical analysis.

### Statistical analysis

Statistical analyses were performed using SAS OnDemand for Academics (SAS Institute Inc., Cary, NC, USA). Descriptive statistics were used to summarize categorical variables, including sex and breed. Continuous variables were assessed for normality using the Shapiro–Wilk test. Variables that were not normally distributed, including age, BW, and body condition score (BCS), were compared between groups using the Mann–Whitney U test.

Normally distributed outcome variables, including lameness score, pain score, thigh circumference, and PVF (% BW), were analyzed using a linear mixed-effects model. Fixed effects included treatment group, time, and the interaction between group and time, whereas individual dogs were included as a random effect to account for repeated measurements.

Least-squares means were estimated to evaluate treatment effects over time. Pairwise post hoc comparisons were performed using the Tukey–Kramer adjustment to compare both within-group changes over time and between-group differences at each evaluation point (months 1, 2, and 3). Estimated mean differences together with their corresponding 95% confidence intervals (CI) were calculated from the least-squares means using the Tukey–Kramer adjustment. The effect size for the PVF outcome was estimated using partial eta-squared (η²p), with a value of 0.16 indicating a large treatment effect. Statistical significance was established at p < 0.05.

## RESULTS

### Baseline characteristics of the study population

A total of nine dogs completed the study. One dog assigned to the UWTM group was withdrawn due to behavioral noncompliance, as it could not be consistently guided across the force plate to obtain valid gait kinetic measurements. Consequently, four dogs in the UWTM group and five dogs in the non-UWTM group completed the entire 12-week study protocol.

The study population comprised three female and six male dogs representing five breeds, including Golden Retriever (n = 3), mixed breed (n = 3), Labrador Retriever (n = 1), Shiba Inu (n = 1), and Siberian Husky (n = 1). FHO was performed on the right hindlimb in seven dogs and on the left hindlimb in two dogs. Individual subject characteristics are presented in Table 4.

Baseline demographic and clinical characteristics are summarized in Table 5. Dogs in the UWTM group had a mean BW of 23.35 ± 8.83 kg, whereas those in the non-UWTM group had a mean BW of 21.29 ± 7.89 kg. The mean age was 2.45 ± 2.75 years and 2.40 ± 1.58 years in the UWTM and non-UWTM groups, respectively. Mean BCS values were 3.0 ± 0.0 and 3.2 ± 0.42 for the UWTM and non-UWTM groups, respectively.

No statistically significant differences were observed between the treatment groups with respect to age, BW, BCS, or the surgical side of FHO (all p > 0.05), indicating that the two groups were comparable before initiation of rehabilitation.

### Clinical outcomes

No dogs in either the UWTM or non-UWTM group experienced major or minor treatment-related adverse events during or after rehabilitation, including skin lesions, water-associated surgical site infections, or panic-related injuries. Following the initial clinical assessment and force plate gait analysis, all dogs exhibited transient exacerbation of lameness that required short-term administration of NSAIDs (carprofen; 4.4 mg/kg orally once daily) for several days. No additional analgesic treatment was required following subsequent evaluation sessions.

Individual clinical outcomes and force plate measurements for all dogs at each evaluation time point are presented in Table 6.

Lameness scores improved progressively throughout the study (Tables 6 and 7). In the UWTM group, lameness scores were significantly lower at both 2 months (mean difference = 1.75; p = 0.02) and 3 months (mean difference = 2.00; p = 0.006) compared with the 1-month postoperative assessment. In contrast, dogs in the non-UWTM group showed no significant improvement between the 1- and 2-month evaluations (p = 0.39), although lameness scores were significantly reduced at 3 months compared with 1 month after surgery (mean difference = 1.40; p = 0.03).

Pain scores did not change significantly over time in the UWTM group (all p > 0.05). Similarly, no significant difference was observed in the non-UWTM group between the 1- and 2-month postoperative assessments (mean difference = 0.60; p = 0.48). However, pain scores were significantly lower at 3 months than at 1 month postoperatively (mean difference = 1.20; p = 0.02).

Thigh circumference of the operated limb remained unchanged throughout the study in both groups, with no statistically significant differences observed between postoperative time points (all p > 0.05).

**Table 4:** Summary of the subjects' characteristics and baseline data.

**Subject**	**Breed**	**Age (years)**	**Sex**	**Weight (kg)**	**BCS**	**Side of FHO**	**Treatment group**
Dog 1	Shiba Inu	7.0	Male	11.8	3	Right	UWTM
Dog 2	Siberian Husky	1.0	Female	20.6	3	Right	UWTM
Dog 3	Mixed breed	5.0	Female	14.3	3	Right	Non-UWTM
Dog 4	Mixed breed	3.0	Male	13.4	3	Right	Non-UWTM
Dog 5	Labrador Retriever	0.8	Male	34.9	3	Right	UWTM
Dog 6	Golden Retriever	1.5	Male	32.9	3	Left	Non-UWTM
Dog 7	Golden Retriever	1.0	Male	26.0	3	Right	UWTM
Dog 8	Golden Retriever	0.5	Female	27.0	4	Left	Non-UWTM
Dog 9	Mixed breed	2.0	Male	18.8	3	Right	Non-UWTM

BCS = Body condition score; FHO = Femoral head and neck ostectomy; UWTM = Underwater treadmill.

**Table 5:** Baseline comparability between the UWTM and non-UWTM groups.

**Parameter**	**UWTM (n = 4)**	**Non-UWTM (n = 5)**	**Statistical test**	**p-value**
Age (years), mean ± SD (range)	2.45 ± 2.75 (0.8–7.0)	2.40 ± 1.58 (0.5–5.0)	Mann–Whitney U test	0.38
Body weight (kg), mean ± SD (range)	23.35 ± 8.83 (11.8–34.9)	21.29 ± 7.89 (13.4–32.9)	Mann–Whitney U test	0.56
BCS, mean ± SD (range)	3.0 ± 0.0 (3–3)	3.2 ± 0.42 (3–4)	Mann–Whitney U test	0.11
FHO surgical side, n (%)			Fisher's exact test	0.44
Right	4 (100)	3 (60)		
Left	0 (0)	2 (40)		

BCS = Body condition score; FHO = Femoral head and neck ostectomy; SD = Standard deviation; UWTM = Underwater treadmill.

### Force plate gait analysis: PVF

Changes in PVF are summarized in Tables 6 and 7. Within the UWTM group, PVF of the operated limb did not differ significantly between the 1- and 2-month postoperative evaluations (mean difference = 12.27; p = 0.19). However, PVF increased significantly at 3 months compared with the 1-month postoperative assessment (mean difference = 23.66; p = 0.008), indicating improved weight bearing capacity following rehabilitation.

In contrast, dogs in the non-UWTM group showed no significant changes in PVF of the operated limb throughout the study period (all p > 0.05).

Interlimb PVF asymmetry also improved in the UWTM group. Although the difference in PVF between the operated and contralateral limbs was not significantly reduced between the 1- and 2-month postoperative evaluations (p = 0.08), a significant reduction was observed at 3 months compared with 1 month (p = 0.02), demonstrating progressive restoration of limb loading symmetry. Conversely, interlimb PVF differences remained unchanged throughout the follow-up period in the non-UWTM group (all p > 0.05).

Although dogs receiving UWTM rehabilitation demonstrated greater improvements in both lameness scores and PVF than dogs in the non-UWTM group at the 2- and 3-month postoperative evaluations, between-group differences did not reach statistical significance (all p > 0.05).

**Table 6:** Comparison of clinical outcomes and gait parameters between the UWTM and non-UWTM groups during the 3-month postoperative follow-up period.

**Parameter**	**UWTM group (Mean ± SD)**	**Non-UWTM group (Mean ± SD)**	**Between-group p-value**
Lameness score at a trot (0–4)			
1 month	3.00 ± 0.82ᵃ	3.20 ± 0.84ᵃ	0.99
2 months	1.25 ± 0.96ᵇ	2.40 ± 0.89	0.45
3 months	1.00 ± 1.15ᵇ	1.80 ± 0.84ᵇ	0.78
Pain score (0–4)			
1 month	2.50 ± 0.58	2.40 ± 0.55ᵃ	0.99
2 months	1.50 ± 0.58	1.80 ± 0.45	0.95
3 months	1.50 ± 0.58	1.20 ± 0.45ᵇ	0.95
Thigh circumference of FHO limb (cm)			
1 month	27.00 ± 3.74	27.60 ± 2.61	0.99
2 months	27.63 ± 4.61	28.60 ± 4.10	0.99
3 months	27.00 ± 4.97	29.00 ± 3.61	0.97
PVF of FHO limb (% BW)			
1 month	41.50 ± 7.19ᵃ	44.36	0.70
2 months	53.77 ± 13.55	37.82 ± 16.75	0.69
3 months	65.16 ± 14.20ᵇ	44.18 ± 20.63	0.44
PVF of non-FHO limb (% BW)			
1 month	69.06 ± 15.67	71.90	0.99
2 months	65.31 ± 12.93	75.15 ± 6.64	0.69
3 months	70.09 ± 8.44	75.11 ± 9.46	0.97
Differential PVF (% BW)			
1 month	27.56 ± 9.16ᵃ	27.54	0.67
2 months	11.55 ± 8.47	37.33 ± 20.79	0.41
3 months	7.11 ± 4.26ᵇ	30.93 ± 28.27	0.49

PVF data were available for only one dog in the non-UWTM group at the 1-month postoperative evaluation; therefore, SD could not be calculated. Different superscript letters (ᵃ, ᵇ) indicate significant differences between postoperative time points within the same treatment group. UWTM = Underwater treadmill; SD = Standard deviation; FHO = Femoral head and neck ostectomy; PVF = Peak vertical force; BW = Body weight.

**Table 7:** Mean differences in clinical and kinetic gait parameters within the UWTM and non-UWTM groups during the 3-month postoperative follow-up period.

**Parameter**	**Phase comparison**	**UWTM group difference**	**95% CI of difference**	**Non-UWTM group difference**	**95% CI of difference**
Lameness score	1 and 2 months	1.75*	0.29 to 3.21	0.80	−0.51 to 2.11
	1 and 3 months	2.00*	0.54 to 3.46	1.40*	0.09 to 2.71
	2 and 3 months	0.25	−1.21 to 1.71	0.60	−0.71 to 1.91
Pain score	1 and 2 months	1.00	−0.20 to 2.20	0.60	−0.47 to 1.67
	1 and 3 months	1.00	−0.20 to 2.20	1.20*	0.13 to 2.27
	2 and 3 months	0.00	−1.20 to 1.20	0.60	−0.47 to 1.67
Thigh circumference of FHO limb (cm)	1 and 2 months	0.63	−2.77 to 1.52	1.00	−2.91 to 0.92
	1 and 3 months	0.00	−2.14 to 2.14	1.40	−3.32 to 0.52
	2 and 3 months	0.63	−1.52 to 2.77	0.40	−2.31 to 1.52
PVF of FHO limb (% BW)	1 and 2 months	12.27	−30.68 to 4.60	6.54	−43.94 to 12.08
	1 and 3 months	23.66*	−42.07 to −6.79	0.18	−50.30 to 5.72
	2 and 3 months	11.39	−27.22 to 4.43	6.36	−20.51 to 7.79
PVF of non-FHO limb (% BW)	1 and 2 months	3.75	−5.87 to 16.37	3.25	−18.85 to 16.44
	1 and 3 months	1.03	−10.64 to 11.59	3.21	−18.80 to 16.49
	2 and 3 months	4.78	−14.75 to 5.20	0.04	−8.87 to 8.97

*indicates a statistically significant difference between postoperative time points. 95% CI = 95% confidence interval; BW = Body weight; CI = Confidence interval; FHO = Femoral head and neck ostectomy; PVF = Peak vertical force; UWTM = Underwater treadmill.

## DISCUSSION

### Principal findings and novelty

This study provides novel evidence that a 10-session UWTM rehabilitation protocol accelerates functional weight bearing recovery following FHO, as demonstrated by objective kinetic improvements that were not observed in dogs receiving conventional postoperative management alone. To our knowledge, this is the first prospective, controlled study to objectively evaluate the efficacy of postoperative UWTM rehabilitation following FHO in dogs using kinetic force plate gait analysis. Unlike previous studies of aquatic therapy that primarily focused on chronic osteoarthritis or generalized orthopedic and neurological disorders [3, 27], the present rehabilitation protocol was specifically designed to facilitate postoperative recovery after FHO. By promoting healthy pseudoarthrosis formation, encouraging early active limb use, and minimizing compensatory gait patterns, this targeted rehabilitation strategy provides a practical approach for improving postoperative functional recovery.

Although dogs undergoing UWTM rehabilitation demonstrated superior functional recovery, improvements should be interpreted in the context of the natural healing process following FHO, as the control group also showed gradual improvement in several clinical parameters over time. Nevertheless, only the UWTM group exhibited a significant increase in PVF of the operated limb between 1 and 3 months postoperatively, accompanied by a significant reduction in interlimb PVF asymmetry. Furthermore, lameness scores improved significantly as early as the second postoperative month in the UWTM group, indicating earlier restoration of functional limb use.

### Mechanisms underlying UWTM-assisted recovery

The observed benefits of UWTM are biologically plausible because its therapeutic effects are mediated by the hydrostatic properties of water, particularly buoyancy and viscosity. Buoyancy reduces effective weight bearing and joint loading, whereas water resistance facilitates controlled muscle activation during movement. Together, these properties may reduce postoperative discomfort, support gait retraining, promote muscle function, and encourage earlier use of the operated limb [3, 27, 28].

These mechanisms are particularly relevant following FHO, in which successful recovery depends on the development of a functional pseudoarthrosis, restoration of limb loading, and prevention of persistent compensatory gait patterns. By enabling active movement with reduced mechanical loading, UWTM may serve as an appropriate transitional exercise between restricted postoperative activity and unrestricted land-based locomotion.

### Objective kinetic assessment of functional recovery

Kinetic gait analysis provides an objective, quantitative assessment of weight bearing function and is considered more reliable than subjective lameness assessment alone [13]. Ground reaction forces reflect the combined forces transmitted through the entire limb during locomotion; consequently, PVF represents overall limb function rather than the contribution of an individual joint. Because the trot is considered the most sensitive gait for detecting kinetic abnormalities [14, 15], all dogs were evaluated under standardized trotting conditions to minimize measurement variability.

Although descriptive comparisons suggested greater restoration of weight bearing capacity in the UWTM group than in the non-UWTM group, incomplete PVF recordings in most control dogs at the 1-month evaluation limited direct statistical comparisons between groups. Nevertheless, the available data consistently indicated faster functional recovery in dogs receiving UWTM rehabilitation. Most notably, the UWTM group demonstrated a significant increase in PVF of the operated limb of 23.66% BW between 1 and 3 months postoperatively (p = 0.008), together with a significant reduction in interlimb asymmetry. These objective kinetic improvements were not observed in the non-UWTM group and provide previously unreported evidence supporting the role of UWTM in accelerating postoperative functional recovery following FHO.

### Comparison with previous studies

The magnitude of functional improvement observed in the present study is consistent with previous reports describing the benefits of aquatic rehabilitation. Twarowska* et al.* [3] reported improved joint ROM following a 10-session UWTM rehabilitation program in dogs with orthopedic and neurological disorders. Similarly, meta-analyses of aquatic exercise in patients with osteoarthritis have demonstrated moderate-to-large improvements in pain and physical function [4, 5].

Although these studies involved different patient populations and clinical conditions, they support the biological plausibility of the present findings and reinforce the clinical relevance of UWTM as an adjunctive rehabilitation modality following orthopedic surgery. Collectively, these preliminary findings suggest that UWTM may accelerate postoperative functional recovery after FHO, although confirmation in larger controlled clinical trials is required.

### Lameness and pain outcomes

The significant improvement in lameness scores observed in the UWTM group between 1 and 2 months postoperatively is consistent with previous studies demonstrating that UWTM improves joint mechanics and facilitates functional recovery [3, 27]. In contrast, pain scores did not differ significantly between postoperative evaluations in the UWTM group.

This discrepancy may reflect the inherent subjectivity of observational pain assessment despite measurable improvements in limb function. Clinical pain scores may also be influenced by temperament, stress, examiner interpretation, and the timing of evaluation. Future studies should therefore complement clinician-based assessments with validated owner-completed clinical metrology instruments, such as the Canine Brief Pain Inventory (CBPI) and Liverpool Osteoarthritis in Dogs (LOAD) questionnaire, which may detect subtle behavioral changes that are difficult to identify during clinical examinations [29, 30].

### Safety and feasibility of the rehabilitation protocol

The rehabilitation protocol employed in this study consisted of 21–25-min UWTM sessions performed once weekly for 10 consecutive weeks. This regimen is comparable with previously published protocols that have demonstrated clinical benefits in dogs with orthopedic and neurological disorders, including intervertebral disk disease, spondylosis, hip dysplasia, and patellar luxation [3]. Previous studies have also shown that even a single hydrotherapy session can improve joint mobility [31].

Despite the relatively long rehabilitation period employed in the present study, only one dog was withdrawn for behavioral noncompliance rather than treatment-related complications. No UWTM-associated wound infection, skin lesion, panic-related trauma, or other adverse event was recorded. These observations support the safety, feasibility, and tolerability of UWTM during the early postoperative period following FHO.

### Muscle morphology and timing of rehabilitation

Although thigh circumference did not increase significantly during the study, this finding is consistent with the prolonged timeline required for skeletal muscle recovery following FHO [9]. Rather than promoting early muscular hypertrophy, UWTM appears to facilitate restoration of functional mobility during the initial postoperative period.

Several factors may explain the absence of measurable muscle hypertrophy. First, the small sample size reduced the statistical power to detect modest treatment effects. Second, tape-based thigh circumference measurements, although clinically validated [32], are less sensitive than imaging modalities such as ultrasonography or computed tomography for detecting subtle changes in muscle volume. Third, the 12-week follow-up period may have been insufficient because measurable muscle hypertrophy following FHO generally requires 6–12 months to develop [33].

Early initiation of physiotherapy has previously been shown to improve functional recovery following FHO [16, 34]. In the present study, UWTM rehabilitation commenced approximately 3 weeks after surgery to allow complete wound healing, minimize the risk of water-borne surgical site infection, and ensure veterinary confirmation of physiological readiness before aquatic exercise [35]. Although this rehabilitation period appeared sufficient to produce meaningful functional improvement [36], it was likely too short to demonstrate measurable increases in muscle mass.

### Clinical implications

The findings of this study support UWTM as a practical, low-impact adjunct to postoperative rehabilitation following FHO in dogs. By promoting earlier restoration of functional weight bearing and improved gait performance, UWTM has the potential to enhance postoperative recovery while minimizing mechanical stress on the healing limb. Nevertheless, widespread implementation in routine veterinary practice remains constrained by logistical and financial considerations. The substantial capital investment required for UWTM equipment limits its availability primarily to referral and specialty hospitals, thereby restricting access in rural and resource-limited settings. Furthermore, completing a 10-session rehabilitation program entails both direct treatment costs and indirect burdens, including repeated transportation and owner time commitments, which may adversely affect treatment compliance. Consequently, more accessible rehabilitation strategies, including land-based or hybrid rehabilitation programs, warrant further comparative evaluation.

### Strengths and limitations

This study has several important strengths. It represents the first prospective, controlled investigation to objectively evaluate postoperative UWTM rehabilitation following FHO using kinetic force plate gait analysis. The standardized and reproducible rehabilitation protocol enhances clinical applicability, while integrating objective kinetic measurements with clinical lameness assessment provides a more comprehensive evaluation of postoperative functional recovery than either method alone.

Several limitations should also be considered when interpreting the findings. Because comparable studies were unavailable for estimating treatment effect size, an a priori sample size calculation based on statistical power could not be performed; therefore, sample size was determined using the resource equation approach [19]. In addition, convenience sampling based on owner consent and patient compliance resulted in a relatively small study population, limiting statistical power and generalizability. The non-randomized, non-blinded allocation of treatment groups based on owner compliance may also have introduced selection bias. Furthermore, incomplete PVF recordings in the non-UWTM group at the 1-month postoperative evaluation precluded calculation of SDs for some variables and limited the robustness of statistical comparisons between groups over time. Although the present findings provide preliminary effect size estimates that may facilitate sample size determination for future studies, they should be interpreted cautiously. Finally, the 3-month follow-up period was insufficient to evaluate long-term muscle hypertrophy or degenerative changes within the pseudoarthrosis, and the single-center study design may limit external validity.

### Future directions

The present study evaluated UWTM as a single rehabilitation modality, without incorporating additional physical therapy interventions commonly used in clinical practice. Combining UWTM with passive ROM exercises, passive stretching, low-intensity laser therapy, or therapeutic ultrasound may provide synergistic benefits and further improve postoperative recovery following FHO [16]. Future investigations should therefore include larger randomized, double-blinded controlled trials to validate the present findings. Based on the variability in PVF observed in this pilot study (SD = 14.20% BW), a post hoc power analysis indicates that a minimum of 12 dogs per group would be required to achieve 80% statistical power at p = 0.05. Future studies should also extend follow-up to 6-12 months to evaluate long-term functional recovery, muscle hypertrophy, and pseudoarthrosis remodeling. In addition, incorporating validated owner-completed clinical metrology instruments, such as the LOAD questionnaire [29] and the CBPI [13], would provide complementary information on functional status and quality of life in the home environment. Finally, studies investigating rehabilitation strategies to enhance wound healing and improve joint mobility before initiating UWTM may facilitate earlier intervention and inform the development of evidence-based postoperative rehabilitation guidelines for dogs undergoing FHO.

## CONCLUSION

This prospective controlled pilot study demonstrated that a standardized 10-session UWTM rehabilitation protocol accelerated functional recovery following FHO in dogs. Dogs undergoing UWTM rehabilitation exhibited significant improvements in lameness scores by 2 months postoperatively and achieved a significant increase in PVF of the operated limb, together with reduced interlimb PVF asymmetry, by 3 months after surgery. In contrast, the non-UWTM group showed more gradual clinical improvement without significant kinetic gains. Although thigh circumference did not increase significantly during the study period, the findings indicate that UWTM primarily facilitates early restoration of functional limb use rather than short-term muscle hypertrophy.

Overall, this study provides preliminary but objective evidence that postoperative UWTM rehabilitation can accelerate functional recovery following FHO in dogs. Although confirmation in larger controlled studies is required, these findings support the incorporation of UWTM into evidence-based postoperative rehabilitation programs to optimize functional outcomes after FHO.

## DATA AVAILABILITY

The supplementary data can be made available from the corresponding author upon request.

## GENERATIVE AI DECLARATION

The authors declare that generative artificial intelligence (AI) tools were used solely to improve language, grammar, and readability during manuscript preparation. All scientific content, data analysis, interpretation of results, and conclusions were developed and verified by the authors. The authors take full responsibility for the accuracy, integrity, and originality of the work presented, and no AI tool was listed as an author.

## AUTHORS’ CONTRIBUTIONS

NM: Conceptualization, methodology, investigation, data curation, and writing–original draft. NK, TS: Conceptualization, supervision, project administration, funding acquisition, methodology, formal analysis, and writing–review and editing. PT, PNg, PNa: Methodology, investigation, and writing–review and editing. SH: Conceptualization, supervision, project administration, methodology, investigation, and writing–review and editing. All authors have read, revised, and approved the final manuscript.

## References

[1] Samoy Y, Van Ryssen B, Saunders J (2016). Physiotherapy in small animal medicine. Vlaams Diergeneeskd Tijdschr.

[2] Millis DL, Gaynor JS, Muir WW (2015). Physical therapy and rehabilitation in dogs. Handbook of veterinary pain management St.

[3] Twarowska J, Strychalski J, Gugołek A (2025). A pilot study on the effects of a 10-session underwater treadmill programme on canine joint range of motion. Animals (Basel).

[4] Song JA, Oh JW (2022). Effects of aquatic exercises for patients with osteoarthritis: systematic review with meta-analysis. Healthcare (Basel).

[5] Wang T, Wang J, Chen Y, Ruan Y, Dai S (2023). Efficacy of aquatic exercise in chronic musculoskeletal disorders: a systematic review and meta-analysis of randomized controlled trials. J Orthop Surg Res.

[6] DeCamp CE, Johnston SA, Déjardin LM, Schaefer S (2015). Brinker, Piermattei and Flo's handbook of small animal orthopedics and fracture repair St.

[7] Schulz K, Déjardin L, Slatter D (2003). Textbook of small animal surgery.

[8] Krystalli A, Sideri A, Kazakos GM, Anatolitou A, Prassinos NN (2023). Contribution to the study of perioperative factors affecting the restoration of dog's mobility after femoral head and neck excision: a clinical study in 30 dogs. Animals (Basel).

[9] Engstig M, Vesterinen S, Morelius M, Junnila J, Hyytiäinen HK (2022). Effect of femoral head and neck osteotomy on canines' functional pelvic position and locomotion. Animals (Basel).

[10] Harper TAM (2017). Femoral head and neck excision. Vet Clin North Am Small Anim Pract.

[11] Buckthorpe M, Pirotti E, Villa FD (2019). Benefits and use of aquatic therapy during rehabilitation after ACL reconstruction - a clinical commentary. Int J Sports Phys Ther.

[12] Chiquoine J, Martens E, McCauley L, Van Dyke JB, Zink C, Van Dyke JB (2018). Aquatic therapy.

[13] Brown DC, Boston RC, Farrar JT (2013). Comparison of force plate gait analysis and owner assessment of pain using the Canine Brief Pain Inventory in dogs with osteoarthritis. J Vet Intern Med.

[14] Voss K, Imhof J, Kaestner S, Montavon PM (2007). Force plate gait analysis at the walk and trot in dogs with low-grade hindlimb lameness. Vet Comp Orthop Traumatol.

[15] Della Valle G, Caterino C, Aragosa F, Micieli F, Costanza D, Di Palma C (2021). Outcome after Modified Maquet Procedure in dogs with unilateral cranial cruciate ligament rupture: evaluation of recovery limb function by use of force plate gait analysis. PLoS ONE.

[16] Khazayee V, Emamjomeh E, Paryani M (2025). The effect of early initiation of physiotherapy on the functional recovery of dogs that underwent femoral head ostectomy. Vet Med Sci.

[17] Meomartino L, Greco A, Di Giancamillo M, Brunetti A, Gnudi G (2021). Imaging techniques in veterinary medicine. Part I: radiography and ultrasonography. Eur J Radiol Open.

[18] Pakgohar A, Mehrannia H (2024). Sample size calculation in clinical trial and animal studies. Iran J Diabetes Obes.

[19] Arifin WN, Zahiruddin WM (2017). Sample size calculation in animal studies using resource equation approach. Malays J Med Sci.

[20] Testa B, Reid J, Scott ME, Murison PJ, Bell AM (2021). The short form of the Glasgow Composite Measure Pain Scale in postoperative analgesia studies in dogs: a scoping review. Front Vet Sci.

[21] Lee WR (2000). The effects of an underwater treadmill physical therapy program on two dogs with osteoarthritis.

[22] Alves ACE (2020). Short-term effects of underwater treadmill therapy on ground reaction forces of canine orthopaedic patients. Lisboa: FMV-Universidade de Lisboa; 2020 [cited 2026 Mar 28]. Available from: http://hdl.handle.net/10400.5/19693.

[23] Gruen ME, Lascelles BDX, Colleran E, Gottlieb A, Johnson J, Lotsikas P (2022). 2022 AAHA pain management guidelines for dogs and cats. J Am Anim Hosp Assoc.

[24] Davidson J, Kerwin S, Millis DL, Levine D (2014). Common orthopedic conditions and their physical rehabilitation. Canine rehabilitation and physical therapy St.

[25] Fahie MA, Cortez JC, Ledesma M, Su Y (2018). Pressure mat analysis of walk and trot gait characteristics in 66 normal small, medium, large, and giant breed dogs. Front Vet Sci.

[26] Levine D, Adamson CP, Bergh A, Millis DL, Levine D (2014). Conceptual overview of physical therapy, veterinary medicine, and canine physical rehabilitation. Canine rehabilitation and physical therapy St.

[27] Bliss M, Terry J, de Godoy RF (2022). Limbs kinematics of dogs exercising at different water levels on the underwater treadmill. Vet Med Sci.

[28] Hodgson H, Blake S, de Godoy RF (2023). A study using a canine hydrotherapy treadmill at five different conditions to kinematically assess range of motion of the thoracolumbar spine in dogs. Vet Med Sci.

[29] Walton MB, Cowderoy E, Lascelles D, Innes JF (2013). Evaluation of construct and criterion validity for the ‘Liverpool Osteoarthritis in Dogs’ (LOAD) clinical metrology instrument and comparison to two other instruments. PLoS ONE.

[30] Brown DC, Boston RC, Coyne JC, Farrar JT (2007). Development and psychometric testing of an instrument designed to measure chronic pain in dogs with osteoarthritis. Am J Vet Res.

[31] Preston T, Wills AP (2018). A single hydrotherapy session increases range of motion and stride length in Labrador retrievers diagnosed with elbow dysplasia. Vet J.

[32] McCarthy DA, Millis DL, Levine D, Weigel JP (2018). Variables affecting thigh girth measurement and observer reliability in dogs. Front Vet Sci.

[33] Off W, Matis U (2010). Excision arthroplasty of the hip joint in dogs and cats. Clinical, radiographic, and gait analysis findings from the Department of Surgery, Veterinary Faculty of the Ludwig-Maximilians-University of Munich, Germany. 1997. Vet Comp Orthop Traumatol.

[34] Dycus DL, Levine D, Marcellin-Little DJ (2017). Physical rehabilitation for the management of canine hip dysplasia. Vet Clin North Am Small Anim Pract.

[35] Mojarradi A, De Decker S, Bäckström C, Bergknut N (2021). Safety of early postoperative hydrotherapy in dogs undergoing thoracolumbar hemilaminectomy. J Small Anim Pract.

[36] Lewis MJ, Thomovsky SA, Moore GE (2023). Adaptation of land treadmill scoring system for underwater treadmill in dogs with thoracolumbar intervertebral disk extrusion. Vet J.

